# Early enteral nutrition and mortality in mechanically ventilated septic patients receiving vasopressors: A retrospective cohort study using the MIMIC-IV database

**DOI:** 10.1371/journal.pone.0337118

**Published:** 2025-11-19

**Authors:** Bo Zou, Fengchan Xi, Tao Gao, Wenkui Yu

**Affiliations:** 1 Department of Critical Care Medicine, Affiliated Hospital of Jiangnan University, Wuxi, China; 2 Women‘s Hospital of Nanjing Medical University(Nanjing Women and Children’s Healthcare Hospital), Nanjing, China; 3 Department of Critical Care Medicine, Nanjing Drum Tower Hospital, The Affiliated Hospital of Nanjing University Medical School, Nanjing, China; First Affiliated Hospital of Soochow University, CHINA

## Abstract

**Background:**

The role of early enteral nutrition (EEN) in septic shock remains unclear. This study aimed to evaluate the association between EEN and clinical outcomes in septic patients requiring vasopressor therapy and invasive mechanical ventilation.

**Methods:**

This retrospective cohort study used the MIMIC-IV database and included adult septic patients receiving vasopressors and mechanical ventilation at ICU admission. EEN was defined as enteral nutrition initiated within 48 hours. The primary outcome was 28-day mortality. Secondary outcomes included ICU and hospital length of stay, and duration of mechanical ventilation. Inverse probability of treatment weighting (IPTW) was used to adjust for baseline confounders. Vasopressor dose was stratified based on the maximum norepinephrine-equivalent dose in the first 48 hours: low (<0.1 micrograms/kg/min), medium (0.1–0.5), and high (>0.5). Multivariable regression models were used to assess associations.

**Results:**

A total of 4,673 patients were included, of whom 997 (21.3%) received EEN. Before weighting, EEN was associated with higher 28-day mortality (21.9% vs. 15.3%). After IPTW adjustment, early feeding remained significantly associated with increased mortality (adjusted odds ratio 1.80; 95% confidence interval, 1.42 to 2.27). In stratified analyses, EEN was associated with increased mortality in the medium-dose (odds ratio 1.66; 95% confidence interval, 1.26 to 2.19, p < 0.001) and high-dose groups (1.90; 1.21 to 2.98, p < 0.001), but not in the low-dose group(1.92, 1.12 to 3.27, p = 0.016).

**Conclusions:**

In critically ill septic patients receiving vasopressors and mechanical ventilation, EEN was associated with increased 28-day mortality, particularly among those receiving medium- or high-dose vasopressor therapy.

## Introduction

Sepsis is a life-threatening condition defined by organ dysfunction resulting from a dysregulated host response to infection. It remains a common and critical challenge in intensive care units (ICUs) worldwide [1]. Septic shock, the most severe manifestation of sepsis, is associated with a mortality rate approaching 40%, and imposes a substantial burden on healthcare systems and resources [2,3]. Microcirculatory dysfunction and tissue ischemia play a key role in the development of organ failure during sepsis, with the gastrointestinal tract being particularly vulnerable. Pathophysiological alterations in the gastrointestinal tract, including impaired perfusion and local ischemia, can lead to dysmotility and increased permeability. Compromised gut function further complicates patient management and is associated with clinical features such as absent bowel sounds, abdominal distension, vomiting, increased gastric residuals, gastrointestinal bleeding, and intra-abdominal hypertension.

Nutritional support is a cornerstone of care in critically ill patients. This gastrointestinal dysfunction poses significant challenges for the initiation and maintenance of nutritional support. Vasopressors, although essential for maintaining adequate perfusion in septic shock, may impair gastrointestinal motility and hinder enteral nutrition(EN) [4,5]. Early enteral nutrition (EEN) may help maintain gut barrier integrity, modulate systemic inflammation, and promote metabolic homeostasis [6]. Accordingly, the Surviving Sepsis Campaign (SSC) guidelines recommend EEN for its presumed physiological benefits [7]. However, guidelines also advise delaying EN until adequate circulatory resuscitation and hemodynamic stability are achieved [8,9].

Despite the proposed physiological benefits, evidence from clinical trials and observational studies regarding EEN remains inconsistent. Some studies have reported no improvement in mortality or secondary infection rates, while others have demonstrated potential survival benefits under specific clinical conditions. A randomized clinical trial found that EEN did not reduce mortality or the risk of secondary infections, but was associated with increased digestive complications compared to parenteral nutrition in shock patients [10]. In contrast, one study observed reduced mortality with EEN among patients with stable hemodynamics who remained on vasopressors [11]. Another analysis suggested that mortality benefits may be limited to patients receiving low to moderate doses of norepinephrine, with no such benefit observed in those on high-dose vasopressors [12]. These findings highlight the uncertainty surrounding the safety and efficacy of EEN in patients requiring vasopressor support. The present study aims to address this gap by evaluating the association between EEN and clinical outcomes in septic patients receiving both vasopressor therapy and mechanical ventilation.

## Methods

### Data source

This study was conducted using data from the Medical Information Mart for Intensive Care IV (MIMIC-IV) database (version 2.0) [13]. The MIMIC-IV project is managed by the Massachusetts Institute of Technology Laboratory for Computational Physiology and contains critical care data for over 40,000 ICU patients admitted to the Beth Israel Deaconess Medical Center (BIDMC) from 2008 to 2019. The MIMIC-IV database is publicly available, and researchers who agree to the data use agreement and have completed “protecting human subjects training” can request access. The MIMIC database was approved by the institutional review boards of the BIDMC (2001-P-001699/14) and the Massachusetts Institute of Technology (No. 0403000206), which granted a waiver of informed consent and approved the data sharing initiative. The data were accessed in August 2024 after completing the required training for MIMIC-IV data use.

### Selection of participants

Adult patients (aged ≥18 years) meeting the Sepsis-3 criteria were eligible for inclusion [1]. Sepsis was diagnosed according to the sepsis-3 criteria; in brief, patients with documented or suspected infection and an acute change in total Sequential Organ Failure Assessment (SOFA) score of ≥ 2 points were considered to have sepsis. Patients were excluded if they stayed in the ICU for less than 72 hours. Vasopressor therapy was defined as the use of dopamine, dobutamine, epinephrine, or norepinephrine within the first 24 hours of ICU admission, as recorded in the medication administration records. To ensure clinical significance, only patients with a cardiovascular component of the SOFA score ≥ 2 were included. This approach helped to identify patients with meaningful vasopressor dependency consistent with sepsis-related circulatory dysfunction. To minimize the inclusion of patients capable of oral intake and to further exclude those with milder illness, we restricted our analysis to patients who required invasive mechanical ventilation within 48 hours of ICU admission. Because patients with severe critical illness might benefit the most from the protective effects of early enteral feeding. These interventions were identified from the medication and procedure records in MIMIC-IV. Additionally, we analyzed only the first ICU stay for patients who were admitted to the ICU more than once. Patients were categorized into the early enteral nutrition (EEN) group if any form of enteral feeding was initiated within 48 hours of ICU admission. Those who did not receive enteral nutrition within this time frame were classified as the non-EEN group.

### Data collection

Structured query language was used to extract data from the MIMIC-IV database. The following baseline variables were collected:

Demographics and Anthropometrics: age, sex, height, and admission body weight.

Comorbidities and Illness Severity: Charlson Comorbidity Index, initial SOFA score, and minimum Glasgow Coma Scale (GCS) score on day 1.

Laboratory and Physiologic Parameters (Day 1): white blood cell (WBC) count, blood glucose (maximum and minimum), serum lactate, hemoglobin, platelet count, blood urea nitrogen (BUN), creatinine, albumin, and the PaO₂/FiO₂ ratio.

Vasoactive Therapy: type of vasoactive agents administered and corresponding dosage within the first 48 hours.

Additionally, we calculated the maximum norepinephrine equivalence (NEE) for each patient. The NEE score is defined as a formula for calculating vasoactive agents with norepinephrine dose (µg/kg/min) as a benchmark, excluding inotropes [14]. To reduce the impact of extreme outliers, we applied winsorization by capping NEE values at 2.0 μg/kg/min, a threshold selected based on clinical plausibility. Extremely high doses (>2.0 μg/kg/min) are rare in clinical practice and typically represent unstable hemodynamic states where enteral nutrition is contraindicated. Winsorization allowed us to preserve the overall sample size while reducing the distorting influence of extreme values.

For subgroup analysis, NEE was categorized into three levels—low (≤0.1 μg/kg/min), medium (0.1–0.5 μg/kg/min), and high (>0.5 μg/kg/min)—based on both clinical rationale and the empirical distribution of the data. The low-dose threshold aligns with the cardiovascular component of the SOFA score, while the upper cutoff (>0.5 μg/kg/min) was around the upper quartile (the 75th percentile approximating 0.5 μg/kg/min) of the dataset. This stratification also ensured sufficient sample sizes across groups to enable stable multivariable and sensitivity analyses. Finally, we recorded the length of the ICU and hospital stay and the 28-day mortality rate.

### Primary and secondary outcomes

The primary endpoint was 28-day mortality. Secondary endpoints included hospital length of stay (LOS), ICU LOS, and duration of mechanical ventilation. Analyses of secondary outcomes were limited to patients who survived beyond day 28 to reduce confounding from early mortality.

### Statistical analysis

Baseline characteristics were summarized as means ± standard deviations (SD) for continuous variables and counts with percentages for categorical variables. Between-group comparisons were conducted using t-tests, Chi-square tests, or Wilcoxon rank-sum tests, as appropriate. LOS outcomes were reported as medians with interquartile ranges (IQR) and compared using the Mann-Whitney U test. Missing baseline data were addressed using multiple imputation via predictive mean matching (PMM). Propensity scores were estimated through logistic regression, and inverse probability of treatment weighting (IPTW) was used to adjust for baseline confounding. The following baseline covariates were included: age, sex, and body mass index (BMI); clinical severity and comorbidity indices including Charlson Comorbidity Index (CCI), SOFA score on day 1, and maximum norepinephrine-equivalent dose (NEE) within the first 48 hours of ICU admission; and first day laboratory parameters including maximum and minimum blood glucose, maximum lactate, minimum PaO₂/FiO₂ ratio, minimum Glasgow Coma Scale (GCS), white blood cell count (WBC), hemoglobin, platelet count, albumin, blood urea nitrogen (BUN), and creatinine. These variables were selected based on clinical relevance and data availability. Covariate balance before and after weighting was assessed using standardized mean differences (SMDs), with SMD < 0.1 indicating adequate balance. Weighted logistic regression models were used to evaluate the association between EEN and 28-day mortality. To address residual confounding, we applied a doubly robust estimation approach. A Cox proportional hazards model was fitted to the stabilized IPTW-weighted sample, with adjustment for key covariates including lactate_max, SOFA score, age, gender, BMI, and Charlson comorbidity index. This method provides valid estimates if either the propensity score model or the outcome model is correctly specified. Analyses were conducted using the svycoxph() function under the survey-weighted design. Secondary outcomes were analyzed using IPTW-weighted linear regression and restricted to 28-day survivors. ICU LOS, hospital LOS and duration of mechanical ventilation were treated as continuous outcomes. Due to their right-skewed distributions and the presence of extreme values, these variables were summarized using medians and interquartile ranges (IQRs). Differences between the EEN and non-EEN groups were compared using the Wilcoxon rank-sum test for unweighted comparisons. After IPTW, we applied weighted quantile estimation to obtain adjusted medians and IQRs. For between-group comparisons, a weighted Wilcoxon rank-sum test was performed using survey-weighted methods to account for the IPTW design. All analyses were restricted to 28-day survivors to avoid immortal time bias. Subgroup analyses were conducted within each vasopressor dose stratum, and interaction terms between EEN and vasopressor group were tested to assess heterogeneity of treatment effect.

All statistical analyses were performed using R (version 4.4.3), and a two-sided p value < 0.05 was considered statistically significant.

## Results

**Fig 1:**
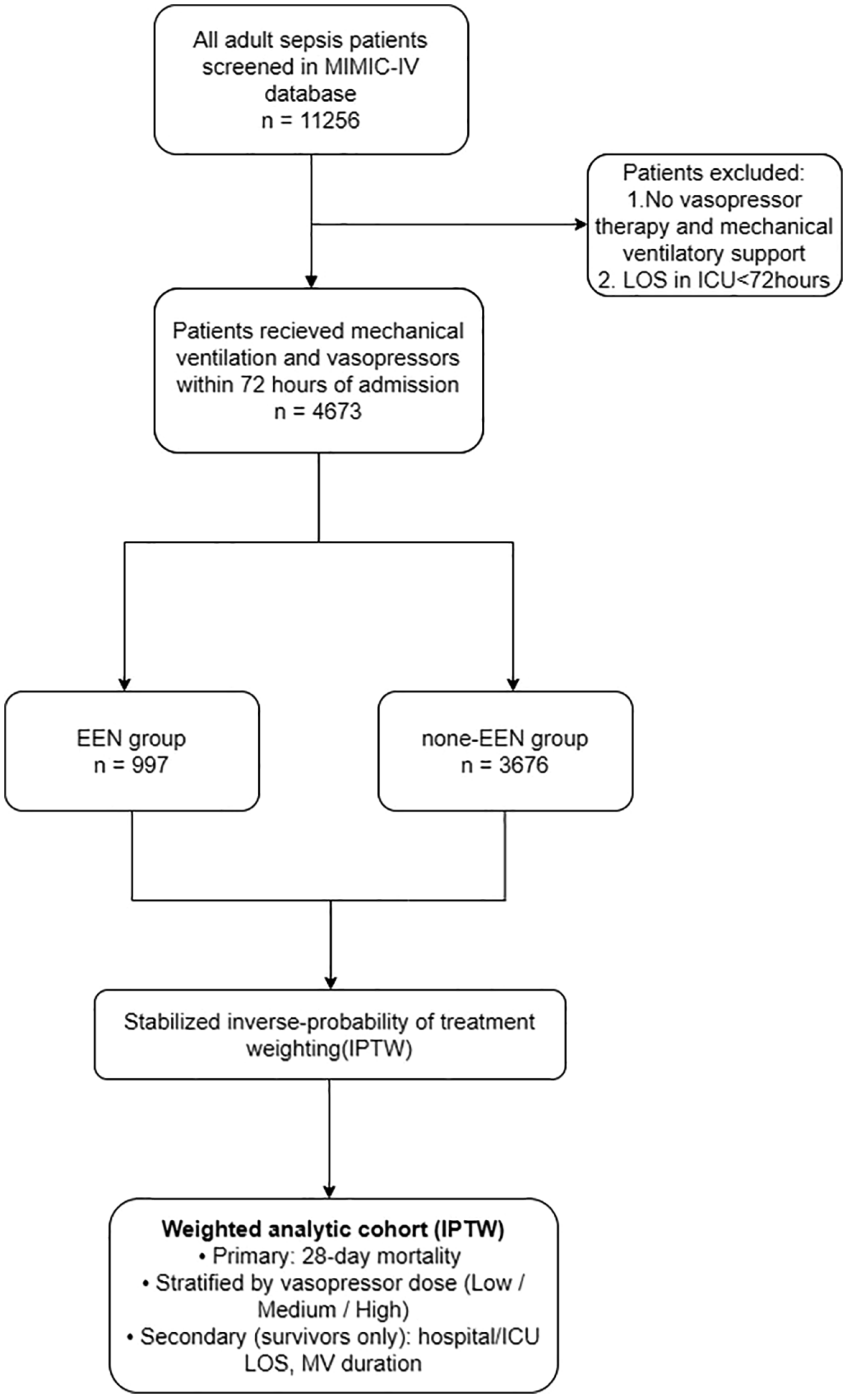
Flowchart of patient selection for the study. MIMIC-IV, Medical Information Mart for Intensive Care IV; EEN, early enteral nutrition; ICU, intensive care unit; LOS, length of stay.

A total of 4673 patients fulfilled eligibility criteria of sepsis 3.0 and received vasopressor and mechanical ventilation therapy within 48 hours after ICU admission, of whom 997 (21.3%) received EEN (Fig 1).

Baseline characteristics of patients in the EEN and non-EEN groups are shown in Table 1. Before IPTW, multiple covariates exhibited significant imbalances between the two groups. Notable differences were observed in sex distribution (59.3% vs. 49.5%, p < 0.001, SMD = 0.197), BMI, SOFA score, NEE, and several laboratory values such as lactate (SMD = 0.459), hemoglobin (SMD = 0.240), and PaO₂/FiO₂ ratio. After IPTW adjustment, covariate balance was substantially improved across most variables. All standardized mean differences (SMDs) were reduced to <0.11, except for lactate (SMD = 0.212) and creatinine (SMD = 0.110), which showed mild residual imbalance. Sex, age, comorbidity index, GCS, platelets, and other key laboratory indices demonstrated excellent balance after weighting, with SMDs well below 0.1 and non-significant p-values.

**Table 1:** Baseline characteristics and severity scoring of the study cohort.

	Before IPTW				After IPTW			
	Non-EEN	EEN	p-value	SMD	Non-EEN	EEN	*P* value	SMD
n	3676	997			3674.38	1045.41		
Male sex, n (%)	2181 (59.3)	494 (49.5)	<0.001	0.197	2105.1 (57.3)	607.8 (58.1)	0.704	0.017
Age, years, mean (SD)	64.51 (14.80)	63.75 (16.02)	0.161	0.049	64.34 (14.83)	63.78 (15.97)	0.431	0.037
BMI, kg/m2, mean (SD)	29.47 (8.20)	30.13 (9.16)	0.029	0.076	29.63 (8.36)	29.72 (8.69)	0.806	0.010
Charlson comorbidity index, mean (SD)	6.00 (2.73)	5.97 (3.02)	0.745	0.011	5.99 (2.72)	5.97 (2.99)	0.866	0.008
SOFA score (mean (SD))	11.40 (3.45)	11.51 (3.18)	0.373	0.033	11.44 (3.42)	11.81 (3.44)	0.038	0.109
Maximum NEE in 48 hours, μg/kg/min, (mean (SD))	0.41 (0.41)	0.38 (0.37)	0.023	0.083	0.41 (0.40)	0.43 (0.40)	0.431	0.038
Laboratory data on day1								
Maximum blood glucose, mg/dL (mean (SD))	200.84 (104.20)	208.83 (106.65)	0.033	0,076	203.01 (106.70)	210.18 (103.18)	0.163	0.068
Minimum blood glucose, mg/dL (mean (SD))	121.11 (45.17)	123.61 (43.70)	0.118	0,056	121.78 (45.71)	121.81 (42.94)	0.987	0.001
Maximum lactate, mmol/L, mean (SD)	4.48 (3.19)	3.14 (2.63)	<0.001	0,459	4.21 (3.05)	4.99 (4.22)	0.01	0.212
Minimum PaO2/FiO2 ratio, mean (SD)	171.62 (97.16)	156.69 (95.65)	<0.001	0,155	168.41 (96.09)	168.73 (101.84)	0.949	0.003
Minimum GCS, mean (SD)	9.77 (4.40)	9.12 (4.08)	<0.001	0,153	9.61 (4.43)	9.31 (4.14)	0.132	0.071
WBC, *10^9/L, mean (SD)	18.53 (12.05)	18.73 (11.30)	0.641	0.017	18.67 (13.46)	19.24 (11.60)	0.366	0.045
Hemoglobin, g/dL (mean (SD))	9.21 (2.19)	9.74 (2.21)	<0.001	0.240	9.33 (2.22)	9.35 (2.26)	0.882	0.008
Platelets, *10^9/L mean (SD)	179.13 (115.54)	199.52 (119.07)	<0.001	0.174	183.21 (116.62)	176.83 (110.92)	0.195	0.056
Albumin, g/dL (mean (SD))	2.83 (0.68)	2.90 (0.67)	0.008	0.096	2.85 (0.68)	2.85 (0.68)	0.898	0.006
BUN, mg/dL mean (SD)	35.52 (24.79)	38.61 (27.63)	0.001	0.118	36.26 (25.59)	37.49 (26.09)	0.289	0.047
Creatinine, mg/dL mean (SD)	1.95 (1.69)	1.94 (1.66)	0.889	0.005	1.95 (1.68)	2.15 (1.99)	0.157	0.110

EEN, early enteral nutrition; BMI, body mass index; SOFA, Sequential Organ Failure Assessment; NEE, norepinephrine equivalent; GCS, Glasgow Coma Scale; PaO₂/FiO₂, partial pressure of arterial oxygen to fraction of inspired oxygen ratio; WBC, white blood cell count; BUN, blood urea nitrogen; SMD, standardized mean difference; IPTW, inverse probability of treatment weighting.

The outcomes of the patients are shown in Table 2. Unadjusted analyses revealed that EEN was associated with a significantly higher 28-day mortality rate compared to the non-EEN group (21.9% vs. 15.3%, Δ = +6.6%, p < 0.001). After applying IPTW, the association between EEN and 28-day mortality remained significant. The IPTW-adjusted odds ratio for mortality was 1.80 (95% CI: 1.42–2.27, p < 0.001). Analyses of ICU LOS, hospital LOS, and ventilation time were applied to patients who survived to day 28. To address potential bias introduced by outliers in LOS distributions, we compared the median and interquartile range (IQR) for both ICU and hospital length of stay (LOS) between EEN and non-EEN survivors using weighted quantile analysis.

**Table 2:** Comparison of Clinical Outcomes Between EEN and Non-EEN Groups: Unweighted and Weighted Analyses.

	Before IPTW		After IPTW	
	EEN effect (EEN vs non-EEN)	p-value	EEN effect(EEN vs non-EEN)	*P* value
28-day mortality, OR (95% CI)	21.9% vs 15.3%	<0.001	OR =1.80 (1.42–2.27)	<0.001
ICU LOS (days) (Median[IQR])	8.59 [5.2–13.6] vs 7.27 [4.6–11.9]	<0.001	8.53 [5.43–13.31] vs 7.29 [4.6–13.01]	0.164
Hospital LOS (days), (Median[IQR])	16.07 [9.7–26.4] vs 15.95 [9.3–25.7]	<0.001	17.60 [11.56–24.61] vs 17.11 [11.05–27.65]	0.494
Ventilation time (hours), (Median[IQR])	17 [10–24] vs 17 [9–23]	0.34	17 [10–24] vs 17 [9–23]	0.246

Note: LOS and ventilation time analyses include 28-day survivors only. EEN, early enteral nutrition; OR, odds ratio; Δ, difference; LOS, length of stay; IPTW, inverse probability of treatment weighting. P-values are derived from weighted Wilcoxon rank-sum tests for post-IPTW comparisons.

For hospital LOS, the median was similar between groups (EEN: 17.6 days [IQR: 11.56–24.61] vs. non-EEN: 17.1 days [IQR: 11.05–27.65]). The difference was not statistically significant (weighted Wilcoxon rank-sum test, p = 0.494). In contrast, for ICU LOS, the EEN group had a slightly higher median (8.53 days [IQR: 5.43–13.31]) compared to the non-EEN group (7.29 days [IQR: 4.60–13.01]), and the difference reached statistical significance (p = 0.00094).

These results suggest that although EEN was associated with a shorter mean hospital LOS in unadjusted models, the median-based comparison shows no meaningful difference in survivors, highlighting the influence of outliers in mean-based estimates.

**Fig 2:**
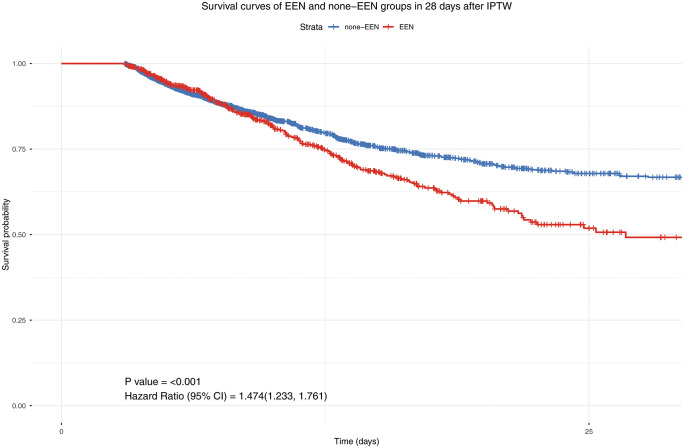
Survival curves of EEN and none-EEN groups in 28 days after IPTW. EEN, early enteral nutrition; IPTW, inverse probability of treatment weighting.

The adjusted hazard ratio (HR) for EEN was 1.47 (95% CI: 1.23–1.76, p < 0.001), indicating a 47% increase in hazard after doubly robust adjustment (Fig 2). Among the covariates, lactate (HR = 1.036, 95% CI: 1.011–1.061, p = 0.0048), SOFA score (HR = 1.042, 95% CI: 1.013–1.072, p = 0.0043), age (HR = 1.013, 95% CI: 1.006–1.020, p < 0.001), and Charlson index (HR = 1.063, 95% CI: 1.030–1.097, p < 0.001) were all significantly associated with increased hazard. Male gender was associated with a reduced hazard (HR = 0.833, 95% CI: 0.716–0.970, p = 0.0188), while BMI was not statistically significant (HR = 0.995, p = 0.31) (Table 3). The model had a moderate discriminatory power, with a concordance index (C-index) of 0.608.

**Table 3:** Doubly Robust Weighted Cox Regression with Stabilized IPTW and Covariate Adjustment.

	HAZARD RATIO (95% CI)	*P* VALUE
**EEN**	1.474(1.233, 1.761)	<0.001
**Maximum lactate**	1.036(1.011, 1.062)	<0.01
**SOFA score**	1.042(1.013, 1.072)	<0.01
**Age**	1.013(1.007, 1.020)	<0.001
**Male (sex)**	0.833(0.716, 0.970)	0.019
**BMI**	0.995(0.984, 1.005)	0.313
**CCI**	1.063(1.030, 1.098)	<0.001

EEN, early enteral nutrition; SOFA, Sequential Organ Failure Assessment; BMI, body mass index; CCI, Charlson comorbidity index, IPTW, inverse probability of treatment weighting.

Patients were stratified into low-dose (n = 737), medium-dose (n = 2656), and high-dose (n = 1280) groups based on maximum NEE dose within 48 hours (cutoffs at 0.1 and 0.5 μg/kg/min). Logistic regression models were adjusted for age, sex, and SOFA score, and weighted using IPTW to minimize confounding. Logistic regression models were adjusted for key clinical covariates, including age, sex, and SOFA score, and further weighted using IPTW to account for baseline confounding. As shown in Table 4, EEN was significantly associated with increased mortality in the medium-dose group (adjusted Odds Ratio(OR) = 1.636 95% CI: 1.26–2.19, p < 0.001) and the high-dose group (adjusted OR = 1.90, 95% CI: 1.21–2.98, p < 0.001). In the low-dose vasopressor stratum, EEN was associated with higher 28-day mortality (adjusted OR = 1.92, 95% CI: 1.22–3.27, p = 0.016).

**Table 4:** Comparisons of adjusted 28-day mortality after stabilized IPTW between EEN and non-EEN groups across vasopressor strata.

Vasopressor dose group	EEN mortality(%)	non-EEN mortality%)	Risk Difference (95% CI)	Odds Ratio (95% CI)	*P* value
Low (≤0.1 μg/kg/min)	16.1	9.1	6.98% (0.49 to 13.48)	1.92 (1.12 to 3.27)	0.016
Medium (0.1–0.5 μg/kg/min)	21.9	14.4	7.44% (2.93 to 11.94)	1.66 (1.26 to 2.19)	0.0003
High(>0.5μg/kg/min)	33.9	21.3	12.66% (2.85 to 22.47)	1.90 (1.21 to 2.98)	0.0047

EEN, early enteral nutrition; IPTW, inverse probability of treatment weighting.

**Fig 3:**
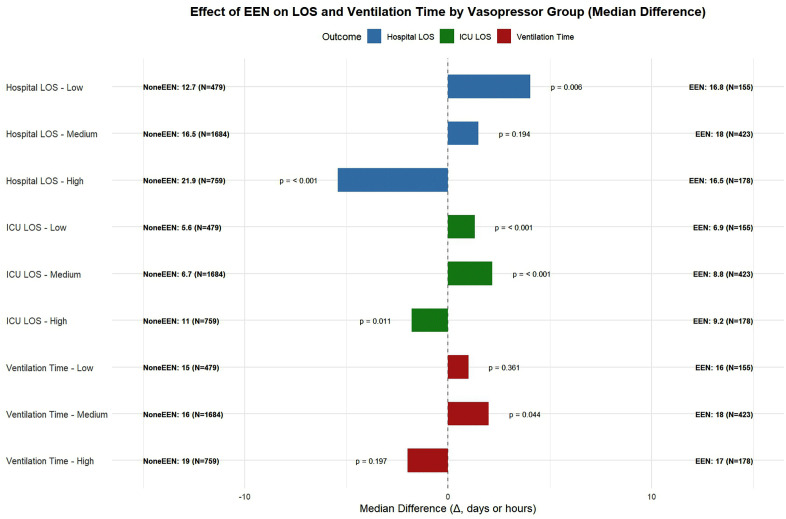
Weighted comparisons of median hospital LOS, ICU LOS, and ventilation time between EEN and non-EEN groups across vasopressor strata among survivors. Median values and p-values are based on IPTW-weighted Wilcoxon rank-sum tests among 28-day survivors. This stratified forest plot, based on Inverse Probability of Treatment Weighting (IPTW) analysis, compares outcomes between patients receiving EEN and those who did not (none-EEN), across three vasopressor dose subgroups. Bars to the right of the dashed line indicate a longer median time for the EEN group. Positive values indicate longer durations in the EEN group, and negative values indicate shorter durations. Low, medium, and high vasopressor dose groups were defined according to the maximum norepinephrine-equivalent dose within the first 48 hours as < 0.1, 0.1–0.5, and > 0.5 μg/kg/min, respectively. Patients number and p values are displayed for each comparison. EEN: early enteral nutrition; LOS: Length of Stay.

We further evaluated the effect of EEN on secondary outcomes stratified by vasopressor dose group among survivors using stabilized IPTW and weighted non-parametric comparisons of medians (Fig 3).

In the high-dose vasopressor group, EEN was associated with a significantly shorter hospital length of stay (LOS) (median: 16.5 vs. 21.9 days; p < 0.001) and shorter ICU LOS (median: 9.2 vs. 11.0 days; p = 0.011), while the difference in ventilation time was not statistically significant (median: 17 vs. 19 hours; p = 0.197).

In the medium-dose group, there was no statistically significant difference in hospital LOS (median: 18.0 vs. 16.5 days; p = 0.194), but EEN was associated with a longer ICU LOS (8.8 vs. 6.6 days; p < 0.001) and slightly longer ventilation time (18 vs. 16 hours; p = 0.044).

In the low-dose group, EEN was unexpectedly associated with a longer hospital LOS (median: 16.8 vs. 12.7 days; p = 0.006) and longer ICU LOS (6.9 vs. 5.6 days; p < 0.001), while ventilation time did not differ significantly (16 vs. 15 hours; p = 0.361). Notably, in the low and medium vasopressor dose groups, EEN was associated with slightly longer ICU stays or ventilation durations, which may reflect survivorship bias or delayed initiation in lower-risk patients.

## Discussion

In this retrospective cohort study of septic patients requiring mechanical ventilation and vasopressor support, EEN was associated with increased 28-day mortality, a finding driven primarily by the subgroups receiving medium or high doses of vasopressors. No survival benefit was observed in the low-dose group. Conversely, among 28-day survivors in the high-dose vasopressor group, EEN was associated with a shorter hospital LOS, a modestly shorter ICU LOS, and no appreciable effect on duration of mechanical ventilation. These findings highlight the need for a more individualized and dose-aware approach to nutritional support in critically ill patients with hemodynamic instability.

Over the past two decades, randomized controlled trials (RCTs) such as EDEN, PERMIT, and TARGET have advanced knowledge on nutritional strategies in critically ill patients. However, these studies primarily enrolled heterogeneous ICU populations and did not specifically address patients with septic shock [15–17]. As a result, current clinical guidelines provide only cautious recommendations for EEN in sepsis. For example, the 2016 American Society for Parenteral and Enteral Nutrition (ASPEN)/ Society of Critical Care Medicine guidelines acknowledged the potential benefits of EEN but highlighted the lack of specific RCTs(9). The 2019 ESPEN guidelines, incorporating evidence from two relevant trials, concluded that early EN may increase gastrointestinal complications without improving mortality or infection rates [8]. The discrepancies among various recommendations may arise from differences in the patient populations studied, as well as the varying definitions and outcomes used to evaluate EEN’s effectiveness. The Surviving Sepsis Campaign(SSC) recommends EEN over early parenteral nutrition (PN) or a combination of EN and PN for patients with shock who are able to tolerate enteral feeding [7]. This recommendation is based on the understanding that EEN supports gut integrity, potentially reduces infection rates, and is associated with better outcomes in critically ill patients. Crucially, during the early acute phase of critical illness, the risk of harm from both starvation and overfeeding is significant. Luis Ortiz-Reyes et al compared EEN to delayed EN in mechanically ventilated patients with circulatory shock, reporting an initial association with improved clinical outcomes that was attenuated after adjustment for illness severity [18].Our study adds to this body of evidence by showing that EEN may be harmful in patients receiving moderate or high doses of vasopressors, a subgroup often underrepresented in major trials. These findings are consistent with the NUTRIREA-2 study, which reported no mortality benefit of EEN but an increased risk of digestive complications in patients receiving high-dose vasopressors (median norepinephrine 0.56 μg/kg/min) [10]. Conversely, a secondary analysis of the EFFORT trial found that EEN was associated with improved outcomes in patients receiving a lower vasopressor dose (median 0.2 μg/kg/min) [18]. Ohbe et al. similarly suggested that EEN may be safe only below a threshold of 0.3 μg/kg/min [12]. Collectively, these findings, including those from the present study, underscore that a fixed vasopressor dose cutoff may not reliably determine EEN safety or efficacy, underscoring the critical need for individualized clinical assessment.

Physiologically, EEN is believed to support gut barrier integrity, preserve mucosal perfusion, and modulate local immunity. However, in patients with compromised splanchnic circulation, such as those receiving high-dose vasopressors, enteral feeding may exacerbate intestinal hypoxia and lead to ischemia. This risk was supported by the higher mesenteric ischemia rate observed in the EEN group of the NUTRIREA-2 trial [10]. Other trials, such as CALORIES, also noted a non-significant trend toward gastrointestinal complications with EN [19]. The ambiguity in defining hemodynamic stability remains a major challenge in clinical practice. Although ESPEN recommends initiating EEN after stabilization, most RCTs defined “early” feeding based on time from ICU admission rather than physiological stability, a discrepancy also noted in the SSC guideline [7]. Several expert groups have attempted to quantify vasopressor burden as a surrogate for circulatory stability. The ASPEN Enteral Nutrition Committee proposed a vasopressor equivalent score, suggesting that a threshold >12 may indicate when EN should be withheld or restricted to trophic feeding [20]. However, this scoring system has not been prospectively validated and may have limited external generalizability. Given the variability in patient responses to EEN, a multifactorial assessment approach may be more appropriate—integrating vasopressor dosage, markers of circulatory stability, and gastrointestinal tolerance to guide the timing and intensity of nutritional support.

Although EEN may confer immunologic and gastrointestinal benefits, it can also impose a metabolic burden during the early phase of critical illness. This period is often marked by mitochondrial dysfunction, characterized by impaired adenosine triphosphate (ATP) production, oxidative stress, and activation of apoptotic pathways. In this context, EEN may increase ATP demand in an already energy-deficient state, potentially worsening outcomes [21]. As oxidative stress rises, mitochondria enter a “hibernation” state, reducing ATP-linked respiration, which worsens organ function and contributes to multiple organ failure. Supporting this hypothesis, a recent prospective cohort study of 1,206 ICU patients found that early nutrition, particularly enteral feeding, was associated with increased 28-day mortality [22]. In the doubly robust weighted Cox model with stabilized IPTW, EEN remained associated with higher 28-day mortality (HR 1.47, 95% CI 1.23–1.76, p < 0.001), suggesting that the signal is unlikely to be explained solely by baseline imbalance. These findings support a more conservative approach to macronutrient delivery during early sepsis, prioritizing essential micronutrients and a delayed, gradual progression toward full caloric targets.

The dose-stratified analyses consistently showed higher 28-day mortality with EEN across all vasopressor strata, with the largest absolute risk differences observed at higher doses. This pattern—wherein harm is magnified at increasing vasopressor intensity—aligns with the pathophysiological expectation that mesenteric hypoperfusion under moderate-to-high vasopressor support may increase the risk of feeding intolerance, interruptions, or ischemic complications, thereby contributing to early mortality.. Previous studies have encountered similar challenges in detecting statistically significant interactions despite observing clinically relevant differences across strata [10,12]. Our findings suggest that while EEN may be tolerated in patients with low vasopressor support, its routine implementation in patients with ongoing hemodynamic instability remains questionable.

Among patients who survived, EEN was associated with a shorter hospital LOS in the high-dose vasopressor stratum and a modestly shorter ICU LOS, whereas the duration of MV showed no appreciable difference across strata. These findings must be interpreted as survivor-specific estimates and do not contradict the primary mortality results. Clinically, these survivor-specific signals suggest that when hemodynamics are sufficiently stabilized—particularly in patients who initially required moderate-to-high vasopressor doses—EEN may coincide with or mark smoother convalescence (shorter ICU/hospital stays), while not meaningfully accelerating ventilator liberation. This underscores a pragmatic approach: prioritize hemodynamic stability, consider trophic starts with close monitoring for intolerance, and recognize that improvements in overall recovery may not translate into shorter MV duration in this population. The apparent tension between the mortality signal and the survivor-only recovery metrics reflects different estimands, not conflicting effects. Mortality is an unconditional endpoint that captures early adverse events, whereas LOS and ventilator days—analyzed among survivors only—condition on post-baseline survival and thus describe the recovery trajectory of those who lived long enough to recover. Conditioning on survival can introduce survivorship (conditioning) bias and should not be read as evidence against the mortality association. Physiologically, under moderate-to-high vasopressor support, mesenteric hypoperfusion and gastrointestinal intolerance make early feeding more likely to precipitate complications, plausibly affecting early survival. By contrast, patients who traverse the unstable phase and tolerate enteral feeding are a selected subset in whom smoother convalescence is expected, which can manifest as shorter stays without implying protection from death.

Our study has several limitations. First, as a retrospective observational analysis, our estimates remain susceptible to residual confounding and model misspecification despite stabilized IPTW and doubly robust adjustment. Second, we did not model achieved EN dose or caloric/target attainment—nor PN dosing—in the primary analyses. These metrics are post-baseline, time-dependent, and may lie on the causal pathway from EEN initiation to outcomes; adjusting for them risks over-adjustment and indication bias, and available EN dose fields were heterogeneous/missing in a way that would have substantially reduced analyzable sample size. Third, the non-EEN group is heterogeneous (e.g., delayed EN, selective PN, or no nutrition), and the timing/route/quantity of nutrition are clinician-driven, time-varying decisions that could influence prognosis and contribute to confounding by indication. Fourth, analyses of length of stay were restricted to survivors, which estimates a survivor-specific outcome and should not be interpreted as contradicting unconditional mortality effects. Finally, vasopressor strata were pre-specified from prior literature (with SOFA-anchored values and quartiles); although exploratory, data-driven thresholds did not materially change conclusions, misclassification around cut-points and limited power to detect formal interaction remain possible. Future research should focus on identifying biomarkers to signal the transition from the catabolic to anabolic phase in critically ill patients, enabling more targeted and individualized nutritional interventions. Additionally, larger, well-designed multicenter trials are needed to evaluate the long-term effects of various nutritional strategies on patient survival, immune function, and recovery.

## Conclusions

In this retrospective cohort study of septic patients requiring vasopressors and mechanical ventilation, EEN was associated with increased 28-day mortality, particularly in those receiving medium to high doses of vasopressors. Among 28-day survivors, EEN was associated with a shorter hospital LOS in the high-dose stratum and a modestly shorter ICU LOS, with no meaningful difference in ventilation duration. These results support a cautious, hemodynamics-informed approach to initiating EEN under vasopressor support. Future prospective studies are needed to determine optimal timing and indications for enteral nutrition based on hemodynamic status and vasopressor dependency.

## Abbreviations

ASPEN: American Society for Parenteral and Enteral Nutrition
ATP: Adenosine Triphosphate
BIDMC: Beth Israel Deaconess Medical Center
BUN: Blood Urea Nitrogen
CCI: Charlson Comorbidity Index
CI: Confidence Interval
EEN: Early Enteral Nutrition
EN: Enteral Nutrition
ESPEN: European Society for Clinical Nutrition and Metabolism
GCS: Glasgow Coma Scale
ICU: Intensive Care Unit
IPTW: inverse probability of treatment weighting
LOS: Length of Stay
MIMIC-IV: Medical Information Mart for Intensive Care IV
NEE: Norepinephrine Equivalence
OR: Odds Ratio
PaO₂/FiO₂: Partial Pressure of Oxygen/Fraction of Inspired Oxygen
PN: Parenteral Nutrition
RCT: Randomized Controlled Trial
SD: Standard Deviation
SMD: Standardized Mean Difference
SOFA: Sequential Organ Failure Assessment
SSC: Surviving Sepsis Campaign
WBC: White Blood Cell.
